# The role of circular RNAs in gastric Cancer: Focusing on autophagy, EMT, and their crosstalk

**DOI:** 10.1016/j.bbrep.2025.102169

**Published:** 2025-07-28

**Authors:** Seyedeh Zahra Bakhti, Saeid Latifi-Navid, Anahita Dah Pahlevan, Latifeh Sarabi, Reza Safaralizadeh

**Affiliations:** a Department of Plant, Cell and Molecular Biology, Faculty of Natural Sciences, University of Tabriz, Tabriz, Iran; b Department of Biology, Faculty of Sciences, University of Mohaghegh Ardabili, Ardabil, Iran; c Cancer Immunology and Immunotherapy Research Center, Ardabil University of Medical Sciences, Ardabil, Iran; d Department of Animal Biology, Faculty of Natural Sciences, University of Tabriz, Tabriz, Iran

**Keywords:** Gastric cancer, Autophagy, EMT, Metastasis, CircRNAs

## Abstract

Globally, gastric cancer (GC) is one of the leading causes of cancer-related mortality. GC is a major threat and concern in human societies because of the high degree of metastasis and the lack of primary diagnostic biomarkers. EMT (epithelial‒mesenchymal transmission) and autophagy through different signaling pathways can regulate GC metastasis. There is evidence that there is an association and crosstalk between EMT and autophagy. EMT-related signaling pathways affect the autophagy process. Conversely, depending on the tissue and stage of the tumor, autophagy has a dual role and can induce/inhibit EMT by modulating different signaling networks. Recent research has shown that circular RNAs (circRNAs) can affect autophagy, EMT, and crosstalk by modulating multiple signaling molecules. Thus, elucidating the interplay between EMT and autophagy and the association of circRNAs with these processes could provide new goals for the identification of biomarkers and the treatment of GC. This review comprehensively discusses the impact of EMT and autophagy on the onset and progression of GC, the functional role of circRNAs as inhibitors/activators, their regulatory mechanisms in regulating autophagy and EMT, and their potential applications as diagnostic biomarkers or anti-GC treatments. Thus, repressing EMT with circRNA-based autophagy inhibitor/activator drugs may be a new strategy that provides insights into the treatment of GC.

## Highlights

• Gastric cancer is a major threat in human societies because of the high degree of metastasis and the lack of primary diagnostic biomarkers.
• Metastasis is a multistage cellular biological process in which EMT and autophagy are involved.
• CircRNAs can modulate EMT, autophagy, and their crosstalk, potentially serving as biomarkers for treatment response.
• Repressing EMT with circRNA-based autophagy inhibitor/activator drugs may be a new strategy for the treatment of GC.

## 1 Introduction

Gastric adenocarcinoma or gastric cancer (GC) ranks fifth in the world in terms of malignancies associated with mortality [1]. Given the absence of biomarkers for early detection and the asymptomatic nature of GC in its initial stages, which leads to late diagnosis, this disease poses a serious social threat and a global concern due to distant metastasis [2]. Metastasis is a multistage process that is regulated by epithelial-to-mesenchymal transition (EMT) and autophagy processes and through various signaling pathways [3]. EMT and autophagy processes are interrelated and are involved in tumor progression [4]. During the EMT process, epithelial cells acquire mesenchymal properties so that they can attack the extracellular matrix. Therefore, the EMT process is essential for the metastasis of tumor cells [5]. Autophagy is an intracellular destruction process that transports damaged components to lysosomes for destruction and thus contributes to energy production and maintenance of cellular homeostasis [6]. However, abnormal autophagy can promote tumor growth and survival [7]. Several studies have shown that autophagy has a positive effect on the development of GC [8,9]. Autophagy plays a crucial role in regulating EMT, suppressing or intensing it depending on the situation. Autophagy prevents the onset of tumors and can inhibit EMT and reverse the EMT phenotype of cancer cells [10]. During tumor progression, autophagy has a positive effect on tumor survival and maintenance after neoplastic lesions [11]. Autophagy promotes tumor cell metastasis through EMT induction [3]. Circular RNAs (circRNAs) are non-coding RNAs derived from back-splicing precursor mRNA transcripts [12,13]. CircRNAs are single-stranded closed loops consisting of a 3′ head attached to the 5′ tail end without a polyadenylated tail and a 5′-3′ polarity. CircRNAs have become a controversial topic due to their special stable structure [14,15]. CircRNAs regulate the expression of various genes at the transcriptional, posttranscriptional, and translational levels by binding to miRNAs or other proteins and molecules [16]. As a result, by changing the expression levels of target molecules and regulating signaling pathways, they play a key role in different types of malignancies [17,18]. Several researches have demonstrated that certain circRNAs show varied levels of expression in GC tissues [19,20]; abnormal levels of circRNAs in GC tumor cells indicate their potential regulatory role in GC initiation and progression [21].

**Fig. 1:**
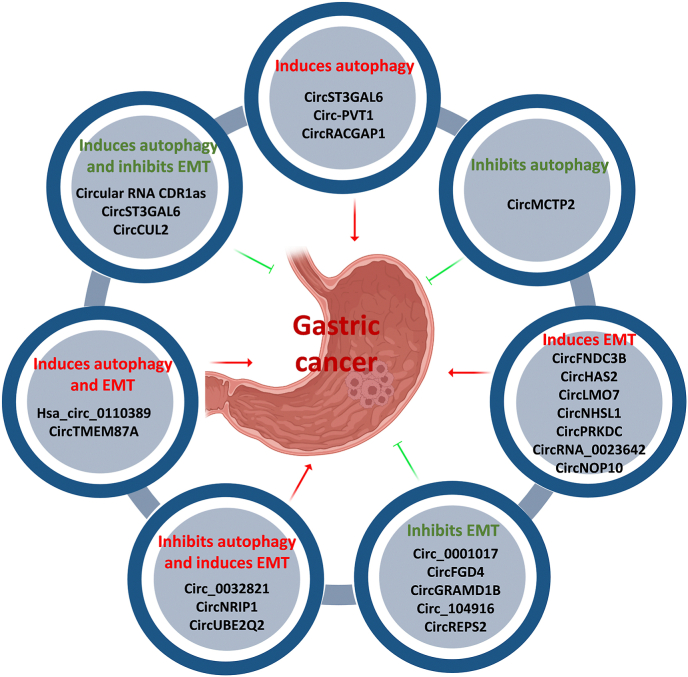
The functions of circRNAs in regulating autophagy, EMT, and crosstalk between them in the suppression or progression of GC.

CircRNAs regulate various cellular activities such as proliferation, apoptosis, autophagy, EMT, migration, invasion, and metastasis, and therefore play a role in carcinogenicity and chemical resistance [22]. Dozens of circRNAs have been discovered to be implicated in the progression of GC. CircHECTD1, circOSBPL10, and circ_0000144 show increased expression in GC and enhance GC cell proliferation, invasion, and migration [23,24]. In addition, circRNAs play crucial roles in the regulation of EMT and autophagy as well as their interference. The TGF-β signaling pathway, a well-known inducer of EMT, has been shown to interact with autophagy mechanisms. TGF-β can promote autophagy in cancer cells, which in turn can enhance EMT. For instance, circRNAs activated by TGF-β have been implicated in promoting tumor metastasis by enhancing the stability of mRNAs involved in EMT [25]. This indicates that circRNAs may serve as mediators of TGF-β signaling, linking autophagy and EMT in the context of GC. Recently, evidence has shown that circRNAs can regulate autophagy and EMT as well as their interactions in the GC; therefore, circRNAs can play an inducer/suppressor role in tumorigenesis (Fig. 1). For example, circ_0032821 [26], circNRIP1 [27], and circUBE2Q2 [28] have been shown to promote tumor progression by suppressing autophagy and inducing EMT in GC cells. Another research found that circTMEM87A promoted GC cell metastasis by inducing autophagy through controlling the miR-142-5p/ULK1 axis [9]. Circular RNA CDR1as can regulate autophagy and EMT via the CDR1as/miR-876-5p/GNG7 axis and thus inhibit metastasis in GC [29].

Hence, comprehensive information on the regulatory function of circRNAs in controlling EMT, autophagy, and crosstalk between them in GC development can be helpful in designing diagnostic and therapeutic strategies. Therefore, our review aims to discuss how autophagy and EMT contribute to GC, and the role of the induction/inhibition of circRNAs and their target molecules and pathways in controlling autophagy, EMT, and their interactions. Finally, the use of circRNAs as biomarkers for GC prognosis and strategies to target circRNAs and their downstream molecules involved in EMT and autophagy processes for GC treatment is discussed.

## 2 Interplay between autophagy and EMT in the progression of GC cancer

Autophagy is a degradation system that transports damaged cytoplasmic components to lysosomes for destruction and energy production for cell renewal and homeostasis [30]. Reports suggest that autophagy functions as a tumor suppressor and prevents tumor onset. However, autophagy can also act as a tumor stimulant and is involved in the progression and maintenance of cancer after neoplastic lesions [31]. Autophagy helps tumor cells overcome hypoxia and nutrient deficiencies by providing substrate and energy. The inhibition of autophagy-related genes causes the death of tumor cells. Autophagy also contributes to the resistance of tumors to chemotherapy [32].

EMT involves the disappearance of epithelial cell characteristics and the emergence of mesenchymal-like cell characteristics, in which different signaling pathways are involved [33]. In EMT, mesenchymal-related molecules such as Vimentin and N-cadherin are expressed more strongly and epithelial-related molecules such as E-cadherin are expressed less strongly. Thus, in EMT, primary tumor epithelial cells do not exhibit cell adhesion or polarity [24]. Abnormal induction of EMT contributes to the initiation of tumor invasion and metastasis to distant parts of the body [34].

As mentioned, both autophagy and EMT are crucial in the progression of tumors, and there is a complicated communication between these two processes. During EMT, tumor cells require the activation of autophagy for energy and survival. However, autophagy can inhibit tumor growth and stop the process of EMT, and the early stages of metastasis [35].

Autophagy influences the EMT process in GC cells. For instance, Qin et al. demonstrated that the inhibition of autophagy promotes metastasis and glycolysis in GC cells through the ROS–NF–κB-HIF-1α pathway, suggesting that autophagy deficiency can facilitate EMT and enhance the metastatic potential [36]. This finding aligns with the observations made by Nie, who noted that increased levels of the autophagy-related protein P62 correlate with diminished autophagic activity, which in turn can promote EMT through the stabilization of mesenchymal markers such as Snail and Twist-1 [37]. The relationship between autophagy and EMT was further underscored by Liang et al., who indicated that uncontrolled autophagy and EMT contribute to malignant phenotypes in GC, highlighting the need for a balanced autophagic response to prevent cancer progression [38].

The role of autophagy in GC is also influenced by various signaling pathways, particularly the PI3K/Akt/mTOR pathway. This pathway is crucial for regulating autophagic flux, and its dysregulation can lead to altered autophagy levels, which in turn affects EMT and cancer cell behavior. For example, Zhao et al. showed that the activation of the PI3K/Akt/mTOR pathway can promote autophagy, thereby influencing the EMT process and the invasive capabilities of GC cells [39]. Furthermore, the work of Wang et al. illustrates that autophagy inhibition can enhance PD-L1 expression in GC, suggesting a potential link between autophagy, immune evasion, and EMT [40]. This highlights the multifaceted role of autophagy not only in cancer progression but also in the immune landscape surrounding tumors.

Moreover, the interaction between *Helicobacter pylori* (*H. pylori*) infection and autophagy presents another layer of complexity in GC development. Courtois et al. reported that autophagy induced by H. pylori is necessary for the emergence of GC stem cells, which are closely associated with EMT and tumor aggressiveness [41]. This relationship suggests that *H. pylori* may exploit autophagic mechanisms to promote EMT and enhance the metastatic potential of GC cells. Additionally, He et al. found that sustained exposure to *H. pylori* lysate inhibits both apoptosis and autophagy in gastric epithelial cells, further complicating the relationship between autophagy, EMT, and tumor progression [42].

## 3 The function of circRNAs in the control of autophagy and GC development

**Fig. 2:**
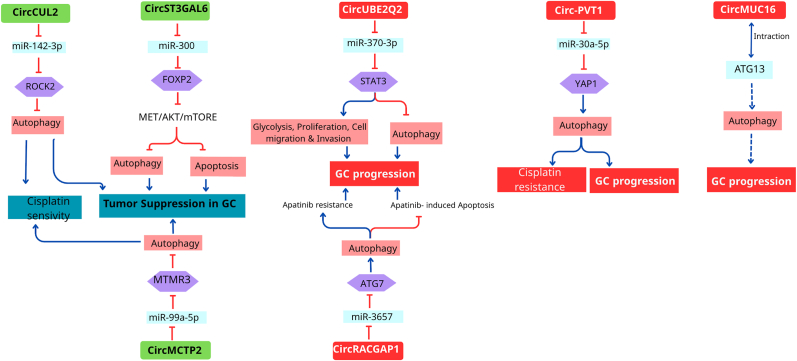
The role of circRNAs as inducers/inhibitors of autophagy in tumorigenesis and chemotherapy resistance of GC. Dashed lines typically indicate a “possible” or “predicted” pathway or component.

Autophagy is involved in tumorigenesis and chemoresistance. In GC, circRNAs have been implicated in modulating autophagy, which is a critical process that can influence tumor growth, metastasis, and response to therapy (Fig. 2). Many studies have shown that autophagy is regulated by circRNAs [43,44]. The overexpression of circST3GAL6 stimulates autophagy and apoptosis in GC cells [45].

CircRNAs are known to function as sponges for microRNAs (miRNAs), thereby regulating the expression of target genes involved in various cellular processes, including autophagy. For instance, Yang et al. demonstrated that circUBE2Q2 promotes malignant progression in GC by regulating signal transducer and activator of transcription 3 (STAT3)-mediated autophagy and glycolysis [28]. This study highlights the role of circRNAs in linking metabolic pathways with autophagy, suggesting that circUBE2Q2 may serve as a potential therapeutic target in GC.

Moreover, circRNAs can directly interact with autophagy-related proteins, thereby influencing the autophagic process. Zhou et al. noted that circRNAs could bind to key proteins involved in autophagy initiation, such as ATG13, which is crucial for the formation of autophagosome [46]. This interaction underscores the potential of circRNAs to modulate autophagy at a mechanistic level, further implicating them in cancer progression. Specifically, circMUC16 promotes autophagy in ovarian cancer through its interaction with ATG13, suggesting that similar mechanisms may exist in GC [44].

The regulation of autophagy by circRNAs is not only limited to direct interactions with autophagy proteins but also involves the modulation of the signaling pathways that govern autophagy. For example, Peng et al. reported that circCUL2 modulated GC progression by inhibiting autophagy through miR-142-3p/ROCK2 pathway in cisplatin-resistant GC cells [47]. This finding indicates that circRNAs can influence the autophagic response to chemotherapy, thereby affecting treatment outcomes in patients with GC.

Circ-PVT1 (circRNA plasmacytoma variant translocation 1) shows high expression levels in GC tissues [48]. Circ-PVT1 stimulated resistance to cisplatin in GC cells by controlling apoptosis, autophagy, and invasion through the regulation of the miR-30a-5p/YAP1 axis. However, the knockdown of this circRNA inhibited resistance to cisplatin by increasing apoptosis and decreasing autophagy and invasion. Circ-PVT1 knockdown increased the P62 level (a proresistance marker) and decreased the LC3-II/I level (a marker of proautophagy), thus inhibiting autophagy. By reducing circ-PVT1, miR-30a-5p expression increased; therefore, the cisplatin resistance of GC was repressed by decreasing the expression level of YAP1 [49].

The level of circMCTP2 expression was decreased in cisplatin-resistant GC cells and tissues. Overexpression of circMCTP2 induces apoptosis and suppresses proliferation and autophagy in GC cells that are resistant to cisplatin. CircMCTP2 directly targets miR-99a-5p and sponges it to upregulate MTMR3, thereby sensitizing GC cells to cisplatin. CircMCTP2 suppresses GC cell resistance to cisplatin by blocking autophagy [50]. MTMR3 can suppress autophagy by functioning as a PI3P phosphatase [51].

CircRACGAP1 regulates autophagy and apatinib sensitivity in GC cells via the miR-3657-ATG7 (autophagy-related gene 7) axis. CircRACGAP1 regulates apatinib-promoted autophagy by targeting and sponging miR-3657 and inhibiting its activity to upregulate ATG7 expression [52].

## 4 The function of circRNAs in controlling EMT and GC development

**Fig. 3:**
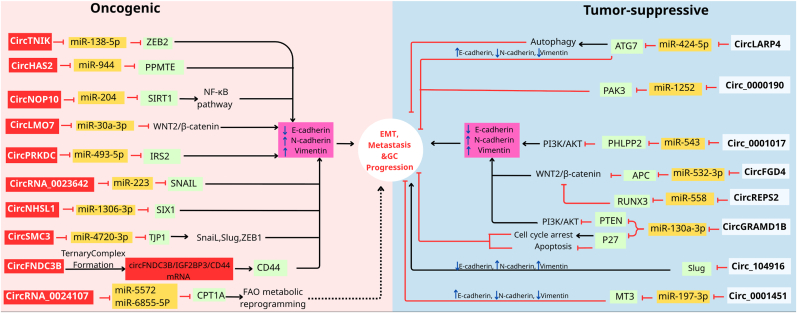
The role of circRNAs as EMT oncogenes/suppressors in GC metastasis and progression. Dashed lines typically indicate a “possible” or “predicted” pathway or component.

EMT is a critical process in cancer progression, enabling epithelial cells to acquire mesenchymal traits, which enhances their migratory and invasive capabilities [53,54]. Various studies have revealed that abnormal circRNA levels in tumor cells are involved in EMT and GC development. Recent research has highlighted the involvement of specific circRNAs in regulating EMT-related pathways. The effects of circRNAs as tumor inducers/inhibitors in EMT regulation and GC progression through the control of signaling pathways are shown in Fig. 3 and Table 1.

**Table 1:** The function of circRNAs as tumor inducers/suppressors in regulating EMT in GC.

CircRNAs	Regulation	Mechanism/s	Function/s	Ref
**CircFNDC3B**	Upregulation	Decrease E-cadherin, and regulates circFNDC3B/IGF2BP3/CD44 mRNA network	Promotes EMT, cell migration, and invasion	[58]
**CircHAS2**	Upregulation	Upregulates PPM1E by targeting hsa-miR-944, and increases Vimentin and N-cadherin expression	Induces tumor cells growth, EMT, migration, and invasion	[59]
**CircLMO7**	Upregulation	Decreases E-cadherin/increases N-cadherin expression, and regulates circLMO7/miR-30a-3p/WNT2/β-Catenin axis	Stimulates tumor cells growth, EMT, migration, invasion, and metastasis	[60]
**CircNHSL1**	Upregulation	Regulates the miR-1306-3p/SIX1/Vimentin axis	Induces the cell motility, invasion, metastasis, and tumorigenesis	[61]
**CircPRKDC**	Upregulation	Decreases E-cadherin/increases N-cadherin expression, and regulates the miR-493-5p/IRS2 axis	Accelerates EMT, migration, invasion, and metastasis capacity	[62]
**CircRNA_0023642**	Upregulation	Regulates the EMT signaling pathway, and alters the expression of EMT-related markers (Vimentin, N-cadherin, and Snail, and E-cadherin)	Induces proliferation, EMT, migration, and invasion	[63]
**CircNOP10**	Upregulation	Regulates the circNOP10-miR204-SIRT1 axis, and NF-κβ pathway	Increases GC cell proliferation, apoptosis, EMT, invasion, and metastasis	[64]
**Circ_0001017**	Downregulation	Reduces Vimentin, and N-cadherin and excesses E-cadherin	Represses EMT, migration, and invasion	[66]
**CircFGD4**	Downregulation	CircFGD4/miR-532-3p axis represses EMT by modulating EMT markers such as Vimentin, N-cadherin, and E-cadherin	Inhibits GC cell growth, migration, EMT, and metastasis	[67]
**CircGRAMD1B**	Downregulation	CircGRAMD1B/miR-130a-3p axis regulated EMT by modulating Vimentin and E-cadherin, and blocked GC by regulating miR-130a-3p-PTEN/p21	Suppresses the ability of GC cells to grow, EMT, migrate and invade	[68]
**Circ_104916**	Downregulation	Downregulates N-cadherin and upregulates E-cadherin, and reduces Slug	Inhibits proliferation, EMT, migration, invasion, and metastasis of GC cells	[69]
**CircREPS2**	Downregulation	Regulates miR-558/RUNX3/β-catenin signaling, and decreases Vimentin and N-cadherin levels, and increases E-cadherin level	Represses the growth, EMT, migration, invasion, and metastasis of GC cells	[70]

CircTNIK promotes tumor progression and metastasis in GC by regulating the expression of ZEB2, a well-known transcription factor that drives EMT [55]. CircRNAs such as circ_0024107 have been linked to lymphatic metastasis in GC, underscoring their relevance in the metastatic cascade [56]. CircSMC3 regulates GC tumorigenesis by targeting the miR-4720-3p/TJP1 axis [57].

CircFNDC3B (fibronectin type III domain‐containing protein 3B) has an oncogenic role and is involved in GC migration, invasion, and progression. CircFNDC3B has been shown to decrease E‐cadherin expression levels and induce EMT in GC. CircFNC3B was correlated with cell adhesion by increasing the level of CD44 expression and forming the circFNDC3B/IGF2BP3/CD44 mRNA network [58].

CircHAS2 functions as an oncogene by increasing PPM1E levels by targeting hsa-miR-944, contributing to the development and advancement of GC. The circHAS2/hsa-miR-944/PPM1E pathway plays a role in promoting growth, EMT, migration, and invasion in GC cells. Reduction of circHAS2 inhibits EMT by decreasing levels of Vimentin and N-cadherin expression [59].

Another oncogenic circRNA is circLMO7 (hsa_circ_0008259), which is increased in GC cells and tissues. The increased expression of circLMO7 led to a reduction in E-cadherin expression and an elevation in N-cadherin expression, thereby stimulating GC cell migration and invasion by inducing the EMT process. CircLMO7 absorbs miR-30a-3p, eliminates its inhibitory effect on WNT2, and can modulate the WNT2/β-catenin signaling pathway to promote growth, EMT, and metastasis in GC cells. In addition, the circLMO7/miR-30a-3p/WNT2/β-catenin pathway provides the energy required for GC cell growth through its effect on glutamine metabolism [60].

A recently identified metastasis-related circRNA called circNHSL1 (hsa_circ_0006835) has an oncogenic function in GC. CircNHSL1 is overexpressed in GC and plays a role in promoting cell metastasis, motility, invasion, and tumorigenesis in GC. Mechanistically, circNHSL1 sponges miR-1306-3p to reduce its inhibitory effect on SIX1 (a transcription factor), so SIX1 intensifies the expression of Vimentin, a mesenchymal marker, to promote EMT, thus inducing motility, metastasis, and invasion in GC cells. Overall, circNHSL1 enhances the advancement of GC through the modulation of the miR-1306-3p/SIX1/Vimentin pathway [61].

A recent study on GC has shown that circPRKDC acts as a tumor inducer and is upregulated in GC samples. CircPRKDC knockdown modulated the levels of EMT markers; after circPRKDC elimination, N-cadherin and E-cadherin levels decreased and increased, respectively. CircPRKDC silencing inhibited GC cell survival, growth, EMT, migration, invasion, and metastasis. CircPRKDC has been shown to accelerate EMT, metastasis, and GC progression by targeting miR-493-5p and positively regulating IRS2 expression [62].

CircRNA_0023642 is a highly regulated circRNA in GC that is associated with proliferation, EMT, migration, and invasion. CircRNA_0023642 regulates the EMT signaling pathway and alters the levels of EMT-associated markers, such as Vimentin, N-cadherin, and Snail, as well as epithelial-associated markers, including E-cadherin [63].

CircNOP10 (hsa_circ-0034351), an oncogenic circRNA, is implicated in the progression of GC and shows increased expression in GC cells and tissues. Mechanically, circNOP10 binds to and sponges miR-204 (a tumor-inhibiting miRNA) to positively regulate SIRT1 expression, leading to increased GC cell proliferation, apoptosis, EMT, metastasis, and invasion by regulating the NF-κβ pathway [64].

Circ_0000190 suppresses GC progression by inhibiting the miR-1252/PAK3 pathway, which is crucial for EMT regulation [65]. Similarly, circRNA circLARP4 has been identified as a tumor suppressor that downregulates EMT markers, thereby inhibiting the invasive potential of GC cells [65].

Circ_0001017, produced from the backsplicing of exportin 1 (XPO1), acts as a tumor inhibitor. Ectopic expression of circ_0001017 represses EMT, migration, and invasion in GC cells. The overexpression of circ_0001017 led to a significant reduction in N-cadherin and Vimentin levels, while increasing E-cadherin levels, ultimately leading to the suppression of EMT [66].

CircFGD4 is a tumor inhibitor of GC development. CircFGD4 is downregulated in GC cells and tissues. Downregulating circFGD4 promotes GC cell proliferation, EMT, migration, and metastasis. CircFGD4 knockdown impacts the expression of EMT associated proteins in GC cells, leading to decreased E-cadherin expression and increased Vimentin expression. CircFGD4 inhibits proliferation, EMT, and cell migration by abrogating cancer-promoting impacts of miR-532-3p. CircFGD4 blocks β-catenin signaling by sponging miR-532-3p and increasing the expression level of APC. The circFGD4/miR-532-3p pathway represses EMT by modulating Vimentin, N-cadherin, and E-cadherin [67].

CircGRAMD1B (hsa_circ_0004798) has an anti-oncogenic role in GC; circGRAMD1B expression is reduced in GC tissues and cells. CircGRAMD1B functions as a sponge for miR-130a-3p, preventing its oncogenic effects and inhibiting the growth, EMT, migration, and invasion of GC cells. The circGRAMD1B/miR-130a-3p axis regulates EMT by modulating Vimentin and E-cadherin. It has also been shown that circGRAMD1B blocks GC tumorigenesis by regulating miR-130a-3p-PTEN/p21 [68].

Circ_104916 functions as an anti-oncogene in GC and is decreased in GC tissues. The overexpression of circ_104916 downregulates N-cadherin and upregulates E-cadherin, thus repressing the EMT process. Circ_104916 inhibits the growth, EMT, migration, invasion, and metastasis of GC cells by decreasing the levels of a transcription factor known as Slug, which inhibits E-cadherin [69].

Another circRNA called circREPS2 acts as a tumor suppressor, with decreased expression in both GC cells and tissues. Mechanistically and functionally, circREPS2 directly targets miR-558 and leads to an increase in the expression of RUNX3 (as a tumor suppressor) to inactivate the Wnt/β-catenin pathway and a decrease in β-catenin levels, thereby suppressing GC progression. High-level expression of circREPS2 affects EMT markers, thereby decreasing the levels of N-cadherin and Vimentin and increasing the level of E-cadherin. CircREPS2 represses the growth, EMT, migration, invasion, and metastasis of GC cells by controlling miR-558/RUNX3/β-catenin signaling [70]. A study showed that hsa_circ_0001451 acts as a sponge for miR-197-3p, thereby targeting MT3 and influencing the malignancy of GC cells. It does so by regulating EMT-associated markers such as, N-cadherin, vimentin, MMP7, and E-cadherin. Consequently, it was found that hsa_circ_0001451 suppresses the proliferation, invasion, and metastasis of GC cells primarily through sponging miR-197-3p and the subsequent regulation of MT3 [71].

MET (mesenchymal-epithelial transition) is the reverse process of EMT, in which mesenchymal cells revert to an epithelial phenotype. Although most research has focused on EMT in cancer progression, recent studies suggest that MET also plays a pivotal role, particularly in metastatic colonization, in which tumor cells revert to an epithelial phenotype [72,73]. The role of circRNAs in the regulation of MET and GC is an emerging area of research highlighting their importance in tumor progression, metastasis, and cellular plasticity. CircRNAs contribute to metastatic colonization by promoting MET, which is a phenotypic change that enables cancer cells to revert to an epithelial state after mesenchymal migration. This switch facilitates tumor colonization at secondary sites, which is crucial in GC metastasis [74]. Although there are no studies that directly investigate the role of circRNAs in facilitating MET and how they affect GC metastasis, dormancy, or treatment response, many studies suggest that circRNAs can also indirectly affect MET by regulating EMT.

## 5 Regulatory effects of circRNAs in controlling the interplay between autophagy and EMT in GC

**Fig. 4:**
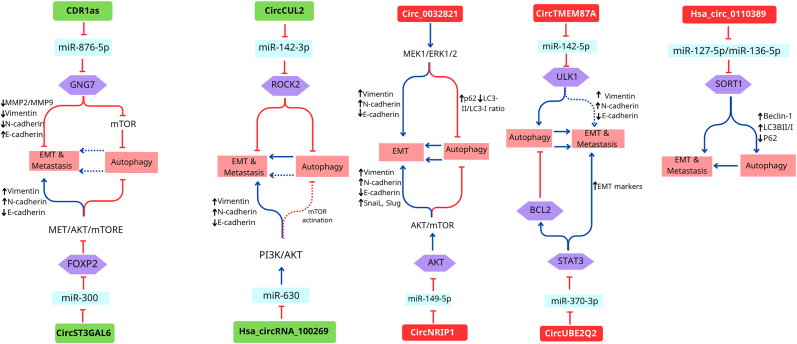
The role of circRNAs as tumor inducers/suppressors in regulating the interaction of autophagy and EMT and GC progression. Dashed lines typically indicate a “possible” or “predicted” pathway or component.

Autophagy can have a dual effect on tumor growth depending on the specific tissue, cell, and tumor stage. Autophagy can enhance EMT by regulating different signaling pathways and providing energy and nutrients for EMT during metastatic release. However, autophagy can suppress EMT and prevent metastasis in the early stages by selectively reducing essential transcription factors involved in EMT. EMT-related signaling pathways affect autophagy [75]. CircRNAs can regulate the interference between autophagy and EMT in GC as oncogenes or suppressors by modulating various signaling pathways (Fig. 4 and Table 2). The interplay between circRNAs, autophagy, and EMT in GC is a burgeoning area of research that underscores the complexity of cancer biology.

**Table 2:** Function of circRNAs in regulation of crosstalk between autophagy and EMT in GC.

CircRNAs	Regulation	Mechanism/s	Function/s	Ref
Circ_0032821	Upregulation	Positively regulates MEK1/ERK1/2 signaling pathway, decreases E-cadherin/increases Vimentin and N-cadherin, and induces the expression of MAPKs, MMP2, and MMP9	Stimulates EMT, cell migration, and invasion, and Inhibits autophagy	[26]
CircNRIP1	Upregulation	Regulates the miR-149-5p-AKT1/mTOR axis	Inhibits autophagy and induces proliferation, EMT, migration, invasion, and metastasis of GC cells	[27]
CircTMEM87A	Upregulation	Regulates the miR-142-5p/ULK1 axis	Induces autophagy and promotes metastasis	[9]
CircUBE2Q2	Upregulation	Regulates the miR-370-3p/STAT3 axis	Inhibits autophagy, enhances glycolysis, and induces EMT, migration, invasion, and metastasis	[28]
Hsa_circ_0110389	Upregulation	Targets miR-127-5p/miR-136-5p and modulates SORT1	Induces autophagy and promotes GC proliferation, migration, invasion, and metastasis	[84]
Circular RNA CDR1as	Downregulation	Regulates the CDR1as/miR-876-5p/GNG7 axis, decreases the expression of the EMT -related proteins, MMP2 and MMP9, and inhibits mTOR signaling	Induces autophagy, and inhibits EMT, and metastasis	[29]
CircST3GAL6	Downregulation	Regulates the miR-300/FOXP2/Met/mTOR axis	Promotes apoptosis and autophagy, and inhibits GC cell growth, invasion, and metastasis	[45]
CircCUL2	Downregulation	Regulates the miR-142-3p/ROCK2	Induces autophagy and represses cell proliferation, migration, invasion, metastasis, and chemical resistance	[47]
Hsa_circRNA_100269	Downregulation	Directly targets miR-630 to PI3K/AKT signaling pathway	Represses proliferation, EMT, migration, invasion, and metastasis of GC cells	[89]

Studies have shown that Hsa_circ_0032821 (circ_0032821) plays an oncogenic role. It is highly expressed in both GC cells and tissues, and is linked to GC malignancy by stimulating EMT, migration and invasion, as well as inhibiting autophagy through positive regulation of the MEK1/ERK1/2 pathway. The upregulation of circ_0032821 facilitates the EMT process by reducing E-cadherin and elevating N-cadherin and Vimentin levels. Additionally, the upregulation of circ_0032821 in GC cells leads to an increase in the levels of MAPKs, MMP2, and MMP9, promoting the migration and invasion of tumor cells. It was also shown in that study that overexpression of circ_0032821 in vitro inhibited autophagy in GC cells through increased p62 and decreased LC3-II/LC3-I ratio. Hence, increased expression of this circRNA can be used as a biomarker for poor prognosis in patients with GC. Silencing or reducing the level of circ_0032821 may have an anticancer effect for treating GC [26].

The circNRIP1, produced by the NRIP gene found on chromosome 21, is formed by splicing together exon II and exon III in a head-to-tail manner. CircNRIP1 contributes to the development of GC as an oncogene by controlling the miR-149-5p-AKT1/mTOR pathway. CircNRIP1 is overexpressed in human GC tissues and acts as a sponge for miR-149-5p to trigger the AKT1/mTOR pathway, thus altering GC cell metabolism and promoting tumor progression. The AKT/mTOR pathway maintains the energy and metabolic homeostasis of tumors by stimulating energy-producing processes and inhibiting catabolic processes such as autophagy. Suppression of autophagy plays an essential role in improved vascular conditions in GC tumors. In addition, the AKT1/mTOR signaling pathway has a positive effect on the EMT process and thus induces GC metastasis. CircNRIP1 suppresses autophagy while promoting growth, EMT, and metastasis in GC cells by sponging miR-149-5p and activating the AKT1/mTOR signaling pathway [27].

CircTMEM87A (circRNA transmembrane protein 87A) has an oncogenic function in GC and is prominently expressed in tumor tissues and cells. This type of circRNA can increase the expression of ULK1 (Unc-51-like autophagy-activating kinase) by sponging miR-142-5p [9]. ULK1 is a crucial component of the autophagy pathway [76] and plays a kinase role that induces autophagy [77]. Therefore, the high expression of ULK1 due to circTMEM87A overexpression leads to increased autophagy, which has a positive effect on the GC promoter. In addition, circTMEM87A has been verified to promote cell metastasis [9]. MiR-142-5p, which represses GC metastasis [78], is sponged by circTMEM87A, thus promoting metastasis. CircTMEM87A may lead to GC progression through the miR-142-5p/ULK1 pathway [9].

Another circRNA that functions as a tumor inducer is circUBE2Q2. Results from a study indicated that circUBE2Q2 is spontaneously upregulated in GC cells and tissues. It sponges miR-370-3p to activate STAT3 (signal transducer and activator of transcription 3) signaling pathway, which leads to suppression of autophagy, induction of glycolysis, upregulation of EMT markers and facilitation of GC metastasis [28]. Improper activation of STAT3 may increase Bcl-2 expression to suppress autophagy, increase glycolysis, and promote cancer cell metastasis, proliferation, and growth [[79], [80], [81], [82], [83]]. The circUBE2Q2/miR-370-3p/STAT3 pathway regulates GC progression by blocking autophagy, increasing glycolysis, and inducing EMT, migration, invasion, and metastasis [28].

Evidence confirms that hsa_circ_0110389 promotes tumor growth, migration, invasion, and metastasis by targeting and binding to miR-127-5p/miR-136-5p and modulating SORT1. In addition, the effects of SORT1 on autophagy may stimulate GC growth and metastasis [84]. Hsa_circ_0110389 knockdown intensifies p62 expression and inhibits LC3B II/I and Beclin-1 expression, which represses autophagy [84].

The circular RNA CDR1as functions as a tumor suppressor in metastasis of GC. The expression levels of the EMT-related proteins MMP2 and MMP9 are negatively regulated by CDR1as. CDR1as sponges miR-876-5p and inhibits its ability to upregulate the expression level of the target gene GNG7 (G protein γ subunit 7) [29]. Research has shown that GNG7 has a suppressive function in tumors [85]. Moreover, GNG7 can cause autophagy by inhibiting mTOR signaling [86]. The circular RNA CDR1as inhibits GC metastasis by regulating the CDR1as/miR-876-5p/GNG7 pathway [29].

A recent study revealed that circST3GAL6 has a tumor-suppressive function in GC. CircST3GAL6 was markedly decreased in the tissues and cells of GC. The overexpression of circST3GAL6 promoted apoptosis and autophagy while suppressing growth, metastasis, and invasion in GC cells. CircST3GAL6 sponges miR-300 and regulates a transcription factor called FOXP2 (forkhead box P2). FOXP2 prevents the Met/AKT/mTOR signaling pathway from being activated [45]. A previous study indicated that the Met/mTOR pathway plays a role in autophagy regulation and stimulation of metastasis [87]. Thus, circST3GAL6 modulates autophagy through its effect on mTOR phosphorylation by inhibiting FOXP2/Met [45]. CircST3GAL6, as a tumor suppressor, also modulates autophagy-mediated proliferation, migration, and metastasis via the miR-300/FOXP2/Met/mTOR pathway [45].

Another circRNA is circCUL2 (hsa_circ_0000234), which is obtained by back-splicing from exon 2 to exon 4, whose expression and function depend on the histological specificity of the circRNA [47]. CircCUL2 causes EMT, tumor metastasis, and tumorigenesis in hepatocellular carcinoma [88]. CircCUL2 is implicated as a tumor suppressor in GC. Its expression is downregulated in the tissues and cells of GC, and its decrease is related to tumor differentiation, TNM stage, and metastasis. The overexpressed circCUL2 likely sponges miR-142-3p to regulate ROCK2 and repress tumor growth, migration, invasion, metastasis, and chemical resistance. Downregulation of circCUL2 cannot block miR-142-3p, so high regulation of miR-142-3p causes malignancy and metastasis in GC tissues. It was also shown that circCUL2 increased cisplatin sensitivity in GC. In addition, circCUL2 could inhibit autophagy via miR-142-3p/ROCK2 in cisplatin-resistant GC cells [47].

Hsa_circRNA_100269 also plays a role in inhibiting GC, and studies have shown that its expression is significantly decreased in GC tissues. Hsa_circRNA_100269 represses growth, EMT, migration, invasion, and metastasis in GC cells by disabling the PI3K/Akt signaling pathway. Elevated levels of hsa_circRNA_100269 led to upregulation of Cas-9, MMP9, Bax and as markers of apoptosis. The overexpression of hsa_circRNA_100269 decreases the level of PI3K; therefore, Akt remains unphosphorylated. Consequently, the levels of the PI3K/Akt downstream target proteins change: p53 levels rise, whereas the levels of cyclin D1 and Bcl-2 decline [89]. A recent study showed that hsa_circRNA_100269 suppresses tumorigenesis by targeting and regulating miR-630 [90].

## 6 Use of circRNAs as EMT and autophagy regulators in GC diagnosis and treatment

Various processes and complex networks of multiple signaling pathways are involved in GC development. Owing to being asymptomatic and lacking early diagnostic biomarkers in the primary stages of GC, the disease is detectable in the advanced stage [91,92]. Recently, despite many advances in diagnosis, chemotherapy, radiotherapy, and surgery, the five-year survival rate for GC patients remains quite low [92]. Identifying the mechanisms and molecules involved in EMT, autophagy, metastasis, and GC development can aid in the discovery of diagnostic molecular biomarkers for the early diagnosis of GC and new therapeutic targets, such as molecular drugs and molecular therapeutic targets, for GC treatment. The clinical implications of circRNAs in GC are significant. Their ability to regulate both autophagy and EMT positions them as potential biomarkers for diagnosis and prognosis. Moreover, targeting circRNAs could provide novel therapeutic strategies to inhibit GC progression by disrupting the crosstalk between autophagy and EMT.

### 6.1 Autophagy- and EMT-related circRNAs as diagnostic biomarkers for the screening and early detection of GC

CircRNAs are characterized by their stable structure and longer half-lives compared with linear RNAs, making them promising candidates for non-invasive biomarkers in cancer diagnostics [93]. The expression profiles of circRNAs in gastric cancer tissues have revealed substantial dysregulation, with numerous circRNAs being either upregulated or downregulated in cancerous tissues compared with normal gastric tissues [94]. Specifically, studies have identified over 300 circRNAs with abnormal expression patterns in GC, suggesting their potential utility as biomarkers for diagnosis and prognosis [71,95]. As mentioned above, changes in the expression levels of circRNAs can significantly affect the modulation of EMT and autophagy processes, resulting in the carcinogenicity of GC. The dual role of circRNAs in both EMT and autophagy presents a unique opportunity to explore their utility as biomarkers that reflect the underlying biological processes driving GC. Moreover, the non-invasive nature of circRNA detection in body fluids such as serum and gastric juice further enhances their appeal as biomarkers for early diagnosis.

Recent studies have highlighted the potential of circRNAs as biomarkers for the early screening and diagnosis/prognosis of GC, particularly in relation to autophagy and EMT processes. One study revealed that circ_0032821 plays an oncogenic role in autophagy, EMT, metastasis, and GC progression by activating the MEK1/ERK1/2 pathway. Therefore, it was suggested that overexpression of circ_0032821 in GC tissues could be a strong biomarker for GC diagnosis [26]. The other two circRNAs involved in the interaction between EMTand autophagy are circTMEM87A and circRNA_100269, which can serve as novel prognostic markers and molecular targets for GC therapy [9,89]. Another biomarker for GC diagnosis is hsa_circ_0110389, which plays a role in GC development. This circRNA is involved in autophagy and metastasis via the miR-127-5p/miR-136-5p-SORT1 axis. Therefore, hsa_circ_0110389 and its downstream molecules could be new therapeutic targets for GC [84]. CircHAS2 and circNOP10 are two oncogenes involved in growth induction, EMT, and GC metastasis and have been indicated to serve as predictive biomarkers for GC. Targeted suppression of circHAS2 and circNOP10, or downstream molecules of these circRNAs, could lead to new therapeutic candidates for the treatment of GC [59,64]. CircST3GAL6 [96] and circFGD4 [67] are two circRNAs that act as tumor suppressors, are downregulated in GC tissues, and inhibit GC metastasis by regulating autophagy or EMT. These circRNAs may be useful factors in diagnosing and treating GC.

Currently, many studies are in the initial discovery and validation phases, aiming to confirm the accuracy and efficacy of circRNAs in smaller clinical cohorts. However, large-scale Phase II or III clinical trials specifically focusing on circRNAs associated with autophagy and EMT as independent diagnostic biomarkers for GC have not yet been reported. Most of these trials are still in the early stages of biomarker validation.

### 6.2 Impact of autophagy and EMT-related circRNAs on the treatment of GC

EMT and autophagy are two important cellular processes in GC development that interact with each other and are regulated by various circRNAs and mechanisms. The role of autophagy and EMT-related circRNAs in the treatment of GC is an emerging area of research that underscores the complexity of cancer biology and the potential for innovative therapeutic strategies. Therefore, strategies targeting circRNAs and their signaling pathways in autophagy and EMT regulation may be new treatment goals to prevent the onset and progression of GC.

For instance, Yin et al. demonstrated that hsa_circ_101882 promotes the migration and invasion of GC cells by regulating EMT markers such as Vimentin and E-cadherin, highlighting the role of circRNAs in facilitating aggressive cancer phenotypes [97]. This suggests that targeting specific circRNAs could potentially reverse EMT and reduce metastasis, offering a novel therapeutic approach. Hsa_circRNA_100269 and circREPS2 act as tumor suppressors in GC, and targeting and inhibiting the hsa_circRNA_100269/PI3K/Akt pathway and circREPS2/miR-558/RUNX3/β-catenin regulatory network can inhibit EMT, metastasis, and GC progression. It has been suggested that targeting hsa_circRNA_100269 and circREPS2 or their downstream molecules could provide new therapeutic approaches for GC management [70,89]. Chen et al. reported that circDLG1 promotes GC progression and resistance to anti-PD-1 therapy by sponging miR-141-3p, which in turn regulates the chemokine CXCL12 [98]. This mechanism illustrates how circRNAs can influence both the tumor microenvironment and the efficacy of immunotherapy, suggesting that circRNA modulation could enhance treatment responses.

The interplay between circRNAs and autophagy in GC has been explored, revealing that certain circRNAs can modulate autophagic pathways, thereby affecting tumor growth and survival [28,45]. For example, circRNA ST3GAL6 has been shown to inhibit malignant behaviors in GC through autophagy regulation, highlighting the potential of circRNAs as therapeutic targets [45]. CircST3GAL6 acts as a tumor inhibitor; therefore, by targeting and increasing its expression, GC can be suppressed by regulating autophagy through the modulation of the FOXP2/MET/mTOR pathway [45].

The intricate relationship between circRNAs, autophagy, and EMT suggests that they may provide novel therapeutic targets or biomarkers for GC management. For example, Liu et al. highlighted that CD146, an EMT marker, correlates with poor prognosis in GC and is associated with autophagy regulation [99]. This indicates that circRNAs involved in the regulation of CD146 may also affect autophagy, thereby influencing tumor progression and treatment outcomes. Furthermore, Wang et al. found that circ_0000190 suppresses GC progression potentially by inhibiting the miR-1252/PAK3 pathway, which is involved in cell migration and invasion [65]. This suggests that circRNAs can serve as potential therapeutic targets to modulate both the autophagy and EMT pathways. CircUBE2Q2 is involved in inhibiting autophagy and EMT induction and promoting GC metastasis. Thus, the overexpression of circUBE2Q2 can be a crucial tumor biomarker and a therapeutic target [28]. CircNRIP1 regulates metabolism and autophagy via the miR-149-5p/AKT1/mTOR axis and induces tumor metastasis. As a result, it has been suggested that targeting and blocking circNRIP1 represents a promising therapeutic approach for managing GC soon [27].

Despite significant advances in understanding the roles of circRNAs, circRNA-based approaches as therapeutics for GC—particularly those targeting autophagy and EMT—are mainly in the preclinical stage (in vitro and in vivo models, such as cell lines and animal studies), and no extensive clinical trials in this field have yet been reported. Most existing studies focus on the molecular mechanisms and initial validation in laboratory and animal models. Many challenges, including effective delivery to target tissues, stability in the body, and treatment safety, need to be addressed. Currently, most clinical trials are centered on more traditional drugs or immunotherapy. However, with further progress, it is expected that circRNA-based strategies will soon enter the early phases of clinical testing. Future research will help clarify the precise roles of these circRNAs and assess their potential for clinical use.

## 7 Challenges, future perspectives, and the road to clinical trials

Despite the promising preclinical evidence, the transition from laboratory research to effective clinical trials remains challenging. In this section, the initial barriers, potential strategies to overcome them, and future directions for circRNA-based diagnosis and treatment in GC are discussed.

### 7.1 Challenges in translating preclinical findings to clinical trials

While circRNAs involved in autophagy and EMT offer promising avenues for diagnosing and treating GC, several limitations and challenges hinder their clinical application. I) Limited understanding of mechanistic roles: The precise molecular mechanisms by which many circRNAs regulate autophagy and EMT in GC remain incompletely understood. Many studies provide correlative data rather than causative evidence, complicating efforts to target circRNAs effectively. II) Technical challenges in detection and quantification: Reliable detection of circRNAs requires sophisticated techniques such as RNase R treatment, qRT-PCR, or sequencing, which may not be standardized or easily available in clinical settings. Variability in sample handling, RNA extraction, and assay sensitivity impacts reproducibility and scalability. III) Limited clinical data and lack of large-scale clinical validation: Although numerous preclinical studies have demonstrated the diagnostic and therapeutic potential of circRNAs, current evidence from large-scale clinical trials is still scarce. Most studies are preliminary, has been performed in vitro, based on cell lines and in animal models, necessitating rigorous clinical validation in human subjects [100]. Large, multi-center clinical trials are required to validate circRNAs as reliable biomarkers or therapeutic targets for GC. IV) Tumor heterogeneity: The heterogeneity of GC—including genetic differences, tumor stage, and microenvironmental factors—significantly influences the expression and regulatory roles of circRNAs and complicates the design of universal diagnostic or therapeutic approaches. CircRNAs are often expressed in a tissue- or disease-specific manner, but overlapping expressions and similar sequences can cause false positives or low specificity in diagnostics. Personalized medicine approaches that account for individual circRNA profiles may help mitigate these issues. Therefore, the utility of circRNAs as biomarkers or therapeutic targets should be tailored specifically to subtype and tumor stage. V) Stability, delivery and targeting: Although circRNAs are generally stable, delivering therapeutic circRNA mimics or inhibitors in vivo remains challenging. Off-target effects, immune responses, and delivery efficiency limit the clinical use of circRNA-based therapies targeting autophagy and EMT pathways. Effective therapeutic interventions targeting circRNAs require precise delivery mechanisms to ensure that therapeutic agents (such as siRNAs, antisense oligonucleotides, or gene therapy constructs) reach the tumor cells in sufficient concentrations without off-target effects. Designing vehicles that can specifically deliver these agents to tumor cells, including those embedded in the TME, is a significant technical challenge [101,102]. VI) Potential off-target and side effects: Targeting circRNAs may inadvertently affect other pathways due to their interactions with multiple miRNAs or proteins [103]. Since autophagy and EMT are involved in normal physiological processes, manipulating circRNAs might lead to unintended adverse effects, such as immune dysregulation or tissue toxicity.

### 7.2 Future perspectives in circRNA-Based clinical trials

Given the compelling preclinical evidence, several promising strategies are emerging to pave the way for clinical trials targeting circRNAs in GC: I) Molecularly Targeted Therapies: CircRNAs such as circCUL2, circUBE2Q2, and etc. Present opportunities for targeted therapies. Future clinical trials could explore the use of synthetic circRNA mimics or the delivery of complementary oligonucleotides (e.g., siRNAs or antisense oligonucleotides) to modulate circRNA levels in tumor cells. For instance, reinstating circCUL2 expression in GC cells may overcome cisplatin resistance by restoring autophagy regulation [47]. II) Combination Therapies: Synergistic therapy combining circRNA modulation with autophagy inhibitors or EMT suppressors could effectively halt tumor progression and metastasis. Combining circRNA-based agents with autophagy inhibitors (e.g., chloroquine) or EMT blockers (e.g., TGF-β inhibitors) could potentiate tumor suppression. For example, silencing oncogenic circRNAs that promote autophagy or EMT may sensitize tumors to chemotherapy or targeted therapies [[104], [105], [106]]. CircUBE2Q2 is known to drive STAT3-mediated oncogenic processes, and preclinical studies have indicated that combining its knockdown with STAT3 inhibitors yields synergistic effects [28]. Future clinical trials could assess the benefits of such combination regimens in patients with advanced GC. III) Liquid Biopsy-Driven Trials: CircRNAs' stability and specificity support their development as non-invasive biomarkers for GC detection and monitoring [93,107,108]. The development of diagnostic assays based on circRNAs holds promise for early detection and treatment monitoring [107]. Integration with other biomarkers (e.g., ctDNA, miRNA) and machine learning algorithms could enhance diagnostic accuracy [109,110]. Clinical trials employing liquid biopsy platforms could evaluate circRNA levels in patient plasma or gastric juice, correlating these with treatment responses and disease progression [111,112]. Such data would be invaluable for stratifying patients and tailoring therapy in a personalized manner [113,114]. IV) Exosome Engineering: With the advent of exosome engineering, researchers are exploring the possibility of loading anti-cancer circRNAs or siRNAs into exosomes for targeted delivery. Engineered exosomes can potentially modulate the tumor microenvironment or directly affect tumor cells, providing a novel therapeutic avenue. Preclinical studies in this area are encouraging and warrant translation into clinical settings [[114], [115], [116], [117]].

### 7.3 Strategies to overcome translational challenges

To facilitate the clinical translation of circRNA-based applications, several strategies should be pursued: Robust Clinical Validation: Large-scale, multicenter studies are needed to validate the diagnostic accuracy and prognostic value of candidate circRNAs. This includes correlating circRNA expression levels with clinical parameters such as tumor stage, metastasis, and patient survival. Advanced Delivery Systems: The development of nanoparticle-based delivery systems and viral vectors specifically engineered to target GC cells will be critical. These delivery platforms must be optimized to protect therapeutic nucleotides from degradation while ensuring targeted cellular uptake. Interdisciplinary Collaboration: Successful translation requires collaboration among molecular biologists, clinicians, bioengineers, and regulatory experts. Integrating expertise from these fields will streamline the development of circRNA-based diagnostic tools and therapeutics. Standardized Assay Development: Establishing standardized protocols for circRNA detection—including isolation, quantification, and normalization—is vital. This will ensure consistency and reproducibility across different clinical laboratories and research centers.

### 7.4 Future clinical trial prospects

Even though current clinical trial data focusing on circRNAs in GC are limited within the available literature, the promising preclinical findings support the initiation of early-phase clinical trials. Such trials could explore: The safety and efficacy of circRNA-targeted therapies (e.g., circCUL2 mimics or inhibitors) in patients with advanced GC. The use of liquid biopsy platforms to monitor circRNA levels as biomarkers for early diagnosis and treatment response. Combination strategies using circRNA modulation alongside conventional chemotherapeutic agents to overcome drug resistance and metastasis. It is anticipated that within the next few years, ongoing research will catalyze the evolution of circRNA-based clinical trials, ultimately leading to novel therapeutic regimens and improved outcomes for patients with GC.

## 8 Conclusion and key insights

GC ranks as the fifth most prevalent cancer associated with fatalities globally and has caused great concern in human societies. In recent years, although many advances have been made in diagnostic methods, chemotherapy, radiation therapy, and surgical procedures, the survival duration of GC patients has remained brief and unsatisfactory [92]. Owing to the distant invasion and metastasis of GC and the lack of early diagnostic biomarkers, GC is still a drastic menace and danger in modern society. Therefore, the discovery of mechanisms involved in GC metastasis, the discovery of diagnostic molecular biomarkers for the initial diagnosis of GC, and the discovery of new therapeutic targets such as molecular drugs and molecular therapeutic targets for GC treatment are essential. CircRNAs have emerged as a groundbreaking class of oncogenic and tumor-suppressive molecules with vast potential in the diagnosis and treatment of gastric cancer. Their inherent stability, resistance to degradation, and ability to regulate key cellular processes—such as autophagy and EMT—make them ideal candidates for targeted therapeutic interventions and non-invasive diagnostic assays.

In this study, we highlighted the role and regulatory processes of circRNAs in GC tumorigenesis. Some circRNAs regulate autophagy or EMT in GC and intensify tumor progression or resistance. CircRNAs such as circ_0032821 [26], circNRIP1 [27], circTMEM87A [9], circUBE2Q2 [28], circNOP10 [64], circRNA_0023642 [63], circPRKDC [62], circNHSL1 [61], circLMO7 [60], circHAS2 [59], and circFNDC3B [58]play oncogenic and tumor aggravating roles, whereas circular RNAs CDR1as [29], circST3GAL6 [45], circCUL2 [47], circREPS2 [70], circ_104916 [69], circGRAMD1B [68], circFGD4 [67], circ_0001017 [66], and circCUL2 [47]act as tumor inhibitors in GC. Therefore, studies have shown that some of these molecules may be useful as diagnostic markers for predicting, early detecting, and treating GC.

Understanding the signaling pathways influenced by circRNAs in regulating EMT and autophagy processes and thus the development of GC can provide a basis for designing new therapeutic strategies and approaches based on circRNAs. Compared with chemotherapy drugs, drugs based on circRNAs do not have severe side effects and do not cause toxicity or cellular immunogenicity. These drugs have specific functions, which is an advantage. The mechanisms and antitumour activities of circRNA-based drugs may be considered. 1) Induction of autophagy by circRNA-based drugs and inhibition of cell growth in the early stages of tumors. 2) Targeting pathways that regulate autophagy-induced EMT or autophagy induction pathways via circRNA-based drugs, thereby inhibiting the growth of tumor cells in advanced tumor stages. Therefore, GC can be counteracted by designing circRNA-based drugs that act as inhibitors or inducers of autophagy. In addition, drugs can be designed that mimic the function of autophagy/EMT-related circRNAs or modulate the function of these circRNAs, thereby treating GC with the help of those drugs. The current preclinical data, including studies on circCUL2, and circUBE2Q2, underscore their roles in modulating chemoresistance, metastasis, and metabolic reprogramming in GC [28,47].

Despite these promising insights, significant challenges remain in translating these findings to clinical practice. The paucity of clinical trial data, the complexity of effective delivery systems, and the intrinsic heterogeneity of GC all represent hurdles that need to be overcome. Nonetheless, the integration of circRNAs into liquid biopsy platforms and the design of novel targeted therapies hold immense promise for the future.

## CRediT authorship contribution statement

**Seyedeh Zahra Bakhti:** Writing – review & editing, Writing – original draft, Supervision, Investigation, Conceptualization. **Saeid Latifi-Navid:** Writing – review & editing, Writing – original draft, Investigation, Conceptualization. **Anahita Dah Pahlevan:** Resources, Investigation. **Latifeh Sarabi:** Resources, Investigation. **Reza Safaralizadeh:** Writing – review & editing, Writing – original draft, Conceptualization.

## Ethical statement

Not applicable.

## Funding

This paper was not funded.

## Declaration of competing interest

The authors declare that they have no known competing financial interests or personal relationships that could have appeared to influence the work reported in this paper.

## Data Availability

No data was used for the research described in the article.
